# Thromboembolism in Older Adults

**DOI:** 10.3389/fmed.2020.470016

**Published:** 2021-01-27

**Authors:** Peter L. Gross, Noel C. Chan

**Affiliations:** Department of Medicine, Thrombosis and Atherosclerosis Research Institute, McMaster University, Hamilton, ON, Canada

**Keywords:** atrial fbrillation, venous thomboembolism, direct-acting anticoagulant, vitamin K antagonist (VKA), falls among older adults, COVID-19

## Abstract

Arterial and venous thromboembolism are both more common in older adults. The use of anticoagulants, the mainstay to prevent thromboembolism, requires consideration of the balance between risk and benefit. Such consideration is even more important in the very elderly in whom the risk of anticoagulant-related bleeding and thrombosis are higher. This review will focus on the challenges of implementing and managing anticoagulant therapy in older patients in an era when the options for anticoagulants include not only vitamin K antagonists (VKAs), but also direct-acting oral anticoagulants (DOACs).

## General Considerations in the Antithrombotic Management of Older Adults

**Figure 1:**
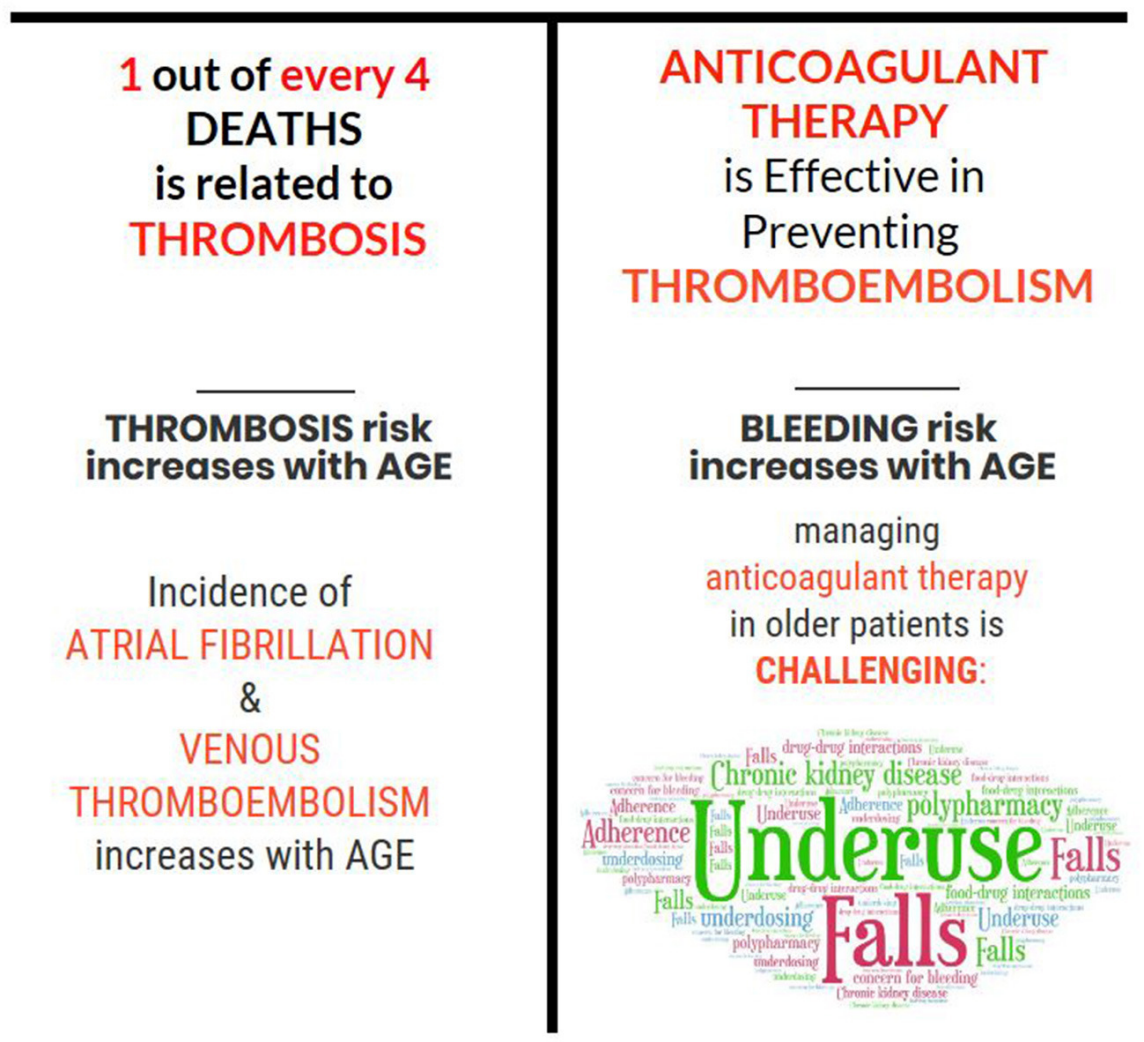
Challenges in managing thromboembolism in older patients.

Thromboembolism is a preventable cause of morbidity and mortality in older patients and the most effective strategy to prevent these outcomes is anticoagulant therapy. Effectively implementing this therapy in older adults is, however, challenging because contraindications and factors that complicate anticoagulation are more prevalent with increasing age (Table 1 and Figure 1). Prevalent features that complicate anticoagulant management in older adults are: non-adherence, falls, chronic kidney disease (CKD), polypharmacy, food-drug, and drug-drug interactions. At a prescriber level, concerns about bleeding have led to the underuse and underdosing of anticoagulants in this population. In this review, we highlight issues that complicate anticoagulation therapy in older patients, discuss up-to-date evidence that will facilitate the assessment of the risk and benefit of anticoagulation therapy, and promote its rational use in older patients with AF or at risk of venous thromboembolism.

**Table 1:** Contraindications to anticoagulant therapy in older patients.

**Absolute contraindication**
Active bleeding
**Relative contraindications**
Amyloid angiopathy Recent intracranial bleed or major bleeding Recurrent GI bleeding not responsive to intervention Severe hypertension Recent major surgery (e.g., neurosurgical) Bleeding diathesis or severe thrombocytopenia

### Adherence

Factors contributing to non-adherence are more common in older patients (1) and non-adherence to a prescribed anticoagulant regimen predisposes to therapeutic failure. Because of differences in the half-lives of VKAs and DOACs, the impact of omitting medications may differ. DOACs have a more rapid offset of action than VKAs (2), and there is concern that missing DOAC doses might result in an inadequate antithrombotic effect more readily than with a VKA. However, the concentration threshold associated with a lack of benefit for each DOAC is unclear (3). On-the-other-hand, VKAs need to be dose adjusted according to the INR. Missing doses is associated with under-anticoagulation and extra doses are associated with over-anticoagulation (4), but missed doses, that a prescriber is unaware of, might lead to inappropriate dose increases, and subsequent over-anticoagulation. For VKA, adherence to treatment not only requires taking the drug but also to INR monitoring and taking the correct dose, a regimen which might not be simple. In the very elderly in whom auditory, visual, cognitive, or mobility limitations are common, the requirement for dose adjustment based on laboratory INR monitoring can be burdensome (5). There is no evidence that the lack of routine laboratory monitoring contributes to decreased adherence or persistence to therapy with DOACs. However, the particular dosing regimens (once daily or twice daily) or food requirement (rivaroxaban needs to be taken with food to optimize absorption) of a DOAC might influence adherence.

### Falls

Falls are more common in the very elderly and are often used as a justification to avoid anticoagulation (6). However, the decision to use or to avoid anticoagulant therapy in such patients needs to take into perspective the risk of harm from falls (particularly the risk of traumatic intracranial bleeding) and the benefit of preventing thromboembolism. Despite the risk of traumatic intracranial hemorrhage, compared with no anticoagulation, observational data suggest a benefit of anticoagulant therapy in older AF patients at risk of falls, who have an estimated annual risk of stroke above 5% (7). Similarly, a modeling study showed that older patients with AF with an additional risk factor for stroke would have to fall 295 times a year for the risk of a subdural hematoma to outweigh the reduction in stroke risk with anticoagulant therapy (8). In a trauma registry of ground-level falls, neither intracranial bleeding nor mortality was higher in patients on VKA than on antiplatelets (9). Also, our ability to predict who will fall and incur bleeding is poor; in one study, those classified as high risk of falls had only a 1.09-fold higher annual risk of bleeding than those classified as low risk of falls (10). Thus, the evidence that anticoagulation causes substantial harm in AF patients with falls is lacking.

### Chronic Kidney Disease

Chronic kidney disease (CKD) is more common in the elderly. Like age, CKD is a risk factor for both thrombosis and bleeding. Although there is a lack of high-quality evidence for anticoagulation in AF patients with severe CKD (estimated glomerular filtration rate (eGFR) <30 ml/min/1.73 m^2^) or end-stage CKD (eGFR <15 ml/min/1.73 m^2^), VKAs have been used in those patients. A meta-analysis of 11 cohort studies showed that compared with no anticoagulation, warfarin was associated with a lower risk of stroke/thromboembolism or mortality without appreciable increase in major bleeding in AF patients with severe CKD (11). In contrast, warfarin was associated with an increase in the risk of major bleeding without reduction in stroke/thromboembolism or mortality in patients with end-stage CKD requiring dialysis. The DOACs have varying degree of renal clearance and as such renal function is a criterion in the selection of dose. In the trials evaluating the DOACs in stroke prevention in AF (SPAF), patients with creatinine clearance (CrCl) <30 ml/min were excluded. Although most regulatory labels indicate a CrCl <15 ml/min as a contraindication for use of a DOAC, most treatment guidelines recommend caution when using DOACs in AF patients in patients with CrCl 15–30 ml/min.

### Polypharmacy

Polypharmacy, defined by the use of multiple medications, is very common in older adults and is associated with increased comorbidity, drug-drug interactions, and worse clinical outcomes. In the pivotal SPAF trials of apixaban and rivaroxaban, polypharmacy was associated with increased thromboembolism, bleeding, and mortality. Therefore, caution is required when managing anticoagulant therapy in older patients. Polypharmacy (defined as ≥5 drugs in the ARISTOTLE trial) was observed in 76.5% of enrolled patients and was more prevalent in older patients. In this trial, patients taking ≥9 concomitant drugs had a 1.5, 1.7, and 2-fold increase in the risk of stroke/SEE, major bleeding, and mortality, respectively, than those taking <5 drugs (12). Likewise, in the ROCKET-AF trial, patients taking ≥10 drugs had a 1.4 and 1.5-fold increase in the risk of major cardiovascular events and clinically relevant bleeding, respectively, when compared with those taking <5 drugs (13). In both trials, the treatment effect of the DOACs vs. VKAs on stroke or systemic embolism was not mitigated by polypharmacy but it diminished the safety advantage of the DOACs.

### Drug-Drug Interactions

Drug-drug interactions are especially relevant in older patients because polypharmacy is common. Cardiovascular drugs, analgesic medications, antimicrobial agents, and drugs acting on the central nervous system are common drug classes that interact with anticoagulants in older patients.

Antiplatelets and non-steroidal anti-inflammatory drugs (NSAIDS) are the most common drugs implicated in adverse drug-drug interactions with anticoagulants. Aspirin increases the risk of bleeding in patients receiving a VKA by 2-fold. For the DOACs, the increased risk of bleeding with concomitant aspirin is 1.3–1.6-fold (14–17). Most guidelines recommend that concomitant aspirin or NSAID use be avoided with anticoagulant therapy, except in circumstances in which there is a strong clinical indication such as after an acute coronary syndrome or after a intravascular stent implantation in the setting of coronary artery or carotid or peripheral artery disease. Concomitant use of NSAIDS is associated with a similar increased risk of bleeding as aspirin and thus should be avoided.

Drug-drug interactions with VKAs include medications that inhibit or induce cytochrome P450 enzymes, contain vitamin K, or alter gastrointestinal flora that metabolize vitamin K (18). Edoxaban and dabigatran are P-glycoprotein substrates, thus drugs that inhibit P-glycoprotein result in higher levels of these anticoagulants. Rivaroxaban and apixaban have a dual mode of clearance, including clearance by efflux pumps such as P-glycoprotein in the kidneys and gastrointestinal system and metabolism by hepatic cytochrome P450-3A4 subtype. Drugs that induce the activity of both P-glycoprotein and cytochrome P450-3A4 result in very low levels of rivaroxaban and apixaban, examples of such medications include: phenytoin, carbamazepine, St. John's Wort and rifampin. The use of rivaroxaban and apixaban with these medications is contraindicated.

### Food-Drug Interactions

Food-drug interactions complicate VKA management, whereby vitamin K-rich foods can quickly reduce the anticoagulant effect. Of the DOACs, rivaroxaban needs to be taken with food for optimal absorption.

### Frailty, Dependency, and Cognitive Function

Randomized prospective studies evaluating the effects of anticoagulants in the elderly likely include subjects with less frailty, dependency, and less cognitive dysfunction than in the real world. Cohort studies that include patients with these factors (19–21) have lower use of anticoagulation. Age and frailty alone should not deter the use of anticoagulation when there is a clinical indication. Both DOACs and warfarin are effective in preventing thrombosis but each has specific advantages and disadvantages which need to be taken into account when selecting an anticoagulant in this population. Advantages of the DOACs include less drug interactions, more simplified dosing and lower risk of intracranial bleeding than warfarin, but some DOACs may have a higher risk of gastrointestinal bleeding. How dependency and cognitive impairment alter the perception of the benefit of anticoagulants in stroke and venous thrombosis prevention in patients, caregivers and prescribers, is not well-studied (22).

## Arterial Thrombosis—Stroke Prevention in Atrial Fibrillation (SPAF)

Atrial fibrillation (AF) is an abnormal cardiac rhythm that increases the risk of stroke by 5-fold (23–26). The incidence of AF increases with age, doubling every decade; it is about 5% a year in those in their 70's and 10% in those in their 80's (27). The case-fatality rate of a stroke with AF is 50% at 1 year, which is double that of a non-cardioembolic stroke (28–30). Similarly, the morbidity of a stroke associated with AF is higher than a non-cardioembolic stroke, 41% of patients with a stroke related to AF are bedridden. Anticoagulant therapy is the most effective strategy to prevent cardioembolic stroke. Thus, compared with placebo or untreated control, VKAs adjusted to an INR range of 2–3 reduce the risk of stroke or systemic embolism by 64%. Shockingly, in an era when VKAs were the only available anticoagulants, anticoagulant use in the elderly declined with increasing age (31, 32); a 5-year increment in age was associated with 0.6 [95% confidence interval (CI) 0.5–0.9] fold reduction of VKA use in patients with AF. Even more astounding is that in optimal environments (single government payer of medical care and medications), anticoagulation in patients with AF is underutilized and up to 50% of eligible AF patients did not receive VKA therapy (33).

The DOACs have been evaluated as alternatives to VKA. Pooled data from 4 large randomized trials indicate that compared to VKAs, DOACs significantly reduce stroke or systemic embolism by 19%, major bleeding by 14% and mortality by 10%. Importantly, irrespective of the degree of INR control, the DOACs had better risk-benefit profile than warfarin (34). Accordingly, several guidelines recommend anticoagulation to prevent stroke in AF and prefer the use of the DOACs over VKAs in most patients (35); a notable exception include AF patients with mechanical heart valve, in whom DOACs are contraindicated and warfarin is still preferred based on data from the RE-ALIGN trial (36). In addition, guidelines currently prefer warfarin over DOACs in AF patients with severe mitral stenosis or with a bioprosthetic valve, conditions that are more prevalent with age, because these patients were underrepresented in the pivotal AF trials. Emerging evidence from a recently completed and from ongoing randomized trials may lead to practice change in the future. Thus, in the recent RIVER trial, that enrolled 1,005 patients with atrial fibrillation and a bioprosthetic mitral valve, rivaroxaban was non-inferior to warfarin with respect to the mean time until the primary composite outcome of death, major cardiovascular events and major bleeding (37).

### The Elderly in the DOAC Trials of SPAF

Four major SPAF trials (38–41) compared the DOACs with VKAs adjusted to an INR range of 2–3. These trials included 22,283 patients aged ≥75, which represented 38% of the overall population. The risk reduction (RR) in stroke and systemic embolism was similar (P-interaction = 0.38) in patients ≥75 years old (RR 0.78; 95% CI: 0.66–0.88) and in those <75 years old (RR 0.85; 95% CI: 0.73–0.99) for the comparison of DOACs vs. VKAs. Likewise, the risk reduction in major bleeding was similar (P-interaction = 0.28) in those ≥75 years old (RR 0.93; 95% CI: 0.74–1.17) and in those <75 years old (RR 0.79; 95% CI: 0.67–0.94) (42). Therefore, older patients in the trials had similar benefit in stroke reduction on a DOAC when compared with warfarin.

In patients with AF who have failed or are unsuitable for warfarin, the AVERROES trial showed that apixaban significantly decreased the risk of stroke or systemic embolism (Hazard Ratio [HR] 0.45; 95% CI: 0.32–0.62) without increasing the risk of major bleeding (HR 1.13; 95% CI: 0.74–1.75) when compared with warfarin (43). In this trial, the absolute rates of stroke or systemic embolism in patients ≥85 were 1.0%/year on apixaban and 7.5%/year on aspirin (HR 0.14; 95% CI 0.02–0.48) and the rates of major bleeding were similar on apixaban and aspirin (4.7% and 4.9%/year) (44). In the recent ELDERCARE-AF trial, that included older Japanese patients with AF (age ≥80 years), compared with placebo, low dose edoxaban (15 mg daily) significantly reduced the rate of stroke or systemic embolism (2.3 vs. 6.7%/year, HR 0.34; 95% CI: 0.19–0.61) without significant increase in the rate of major bleeding (3.3% vs. 1.8%/year, HR 1.87; 95% CI: 0.90–3.89) (45). These findings highlight that older patients with AF remain at high risk of stroke if untreated or given aspirin. Because older patients have higher baseline ischemic risk (46), they stand to benefit the most from the use of an anticoagulant (47–52).

### Practical Considerations in Dosing DOACs for SPAF in the Elderly

Age is an independent criterion for dose adjusting dabigatran and apixaban (53). For apixaban, age ≥80 is one criterion (the others being weight ≤60 kg and serum creatinine ≥133 mM) for selection of the 2.5 mg BID over the 5 mg BID. In the RE-LY trial, compared to younger patients, those aged ≥80, or ≥75 who had an additional bleeding risk factor, had a higher risk of bleeding on the 150 mg BID dose, so such patients are usually given the 110 mg BID dose, where it is available, or 75 mg BID in the US for Cockroft-Gault creatinine clearance (CrCl) between 15 and 30 ml/min. Both edoxaban and rivaroxaban have recommended dose reductions if the CrCl is under 50 mL/min. Age is an important factor in the CrCl calculation. Thus, the usual dose of edoxaban is 60 mg daily, but is reduced to 30 mg daily for CrCl between 15 and 50 ml/min and the usual dose of rivaroxaban is 20 mg daily but is reduced to 15 mg for CrCl between 15 and 50 ml/min. It is important that the labeled dosing of the DOACs, although complicated, be followed to minimize the risk of DOAC under- or overexposure. Post-marketing studies have reported a high prevalence of underdosing, particularly with apixaban (54–56). Off-label dosing has been associated with inferior efficacy (57, 58). Like the results of the phase 3 trials, observational studies showed that the DOACs are at least as effective as VKA and are associated with less intracranial hemorrhage in older patients but some DOAC regimens have been associated with a higher risk of gastrointestinal bleeding. Therefore, caution is required when selecting a DOAC in those at risk of GI bleeding (59, 60).

### The Case to Continue VKA in a Stable Patient

An open question is whether to switch an older person who is optimally anticoagulated with a VKA (with an excellent time in therapeutic range [TTR]) to a DOAC. Most of the patients in the major DOAC SPAF trials enrolled subjects who were new to anticoagulation. Excellent TTR is associated with better outcomes (61). Thus, it might be reasonable for a patient with an excellent TTR to remain on VKAs (48). It is impossible to match subjects with excellent TTR on VKA to another subject receiving a DOAC. In the major DOAC trials, center TTR, which is the average TTR of patients in that center, correlated inversely with bleeding and ischemic events (62–64). Although one of the reports matched patients with good TTR on VKA with DOAC patients and found that the DOAC benefits remain (64).

## Venous Thromboembolism in the Elderly

Venous thromboembolism (VTE), which includes deep vein thrombosis (DVT) and pulmonary embolism (PE), occurs in about 1 in 1,000 persons each year. Incidence rises with age to at least 5 in 1,000 persons in those aged ≥80 (65). Less people present with PE, than DVT alone. Within 1 month of diagnosis, death occurs in ~6% of patients with DVT and 12% of those with PE (66).

Physiological changes in the hemostatic system, such as increasing levels of procoagulant factors (factor VIII, factor VII, and fibrinogen) together with impairment in the fibrinolytic pathway, that occur with aging contribute to the higher risk of VTE in older patients (67). In addition, acquired risk factors, such as cancer and chronic inflammatory disease, are more common and accentuate the risk of VTE in older patients. Not surprisingly, about two-thirds of all VTE events occur in patients over 70 years of age (68).

Acquired risk factors may be found about 50% of patients with VTE and can be categorized into those that are persistent or transient as well as those that are major or minor. Examples of major transient risk factors are surgery, trauma and hospitalization for acute medical illness. With the expanding coronavirus disease (COVID) 2019 pandemic, which has already affected millions of people globally, hospitalization with severe acute respiratory syndrome coronavirus-2 (SARS-CoV-2) is becoming a topical and common acquired risk factor for VTE, particularly in older patients who have higher risks of severe illness, respiratory failure requiring ICU admission, and death (69).

Emerging data indicate that in hospitalized patients with COVID-19, the rates of VTE are high, with estimates ranging from 4.8 to 33.7% despite prophylactic anticoagulation (70–72). The highest VTE rates occur in older patients, in whom the reported rates may be about 1.5–2-fold higher (73). Because of the high VTE risk, many physicians are calling for intensified prophylactic anticoagulation or the empiric use of therapeutic anticoagulation in hospitalized patients with severe COVID-19, but intensifying anticoagulant therapy could result in an even higher risk of bleeding, particularly in critically ill older patients. In a multicenter retrospective study of 400 hospitalized patients with COVID-19 who were receiving prophylactic doses of anticoagulant, the rate of major bleeding was already high, at about 5.6%, and in those with bleeding risk factors such as thrombocytopenia, the corresponding rate was 3-fold higher (70, 72).

Guidance statements from the International Society on Thrombosis and Haemostasis (ISTH) discourage the use of treatment-dose heparin for primary VTE prevention, and are emphasizing the need for universal prophylaxis with standard-dose UFH or LMWH in hospitalized COVID-19 patients, and suggest a 50% increase in the dose of anticoagulant prophylaxis in critically ill patients at the highest risk of VTE or in obese patients in the absence of bleeding contraindications but there is no specific recommendation based on age (74). Ultimately, identifying the optimal approach to prevent VTE in older patients with COVID-19 requires evaluation in randomized trials.

**Figure 2:**
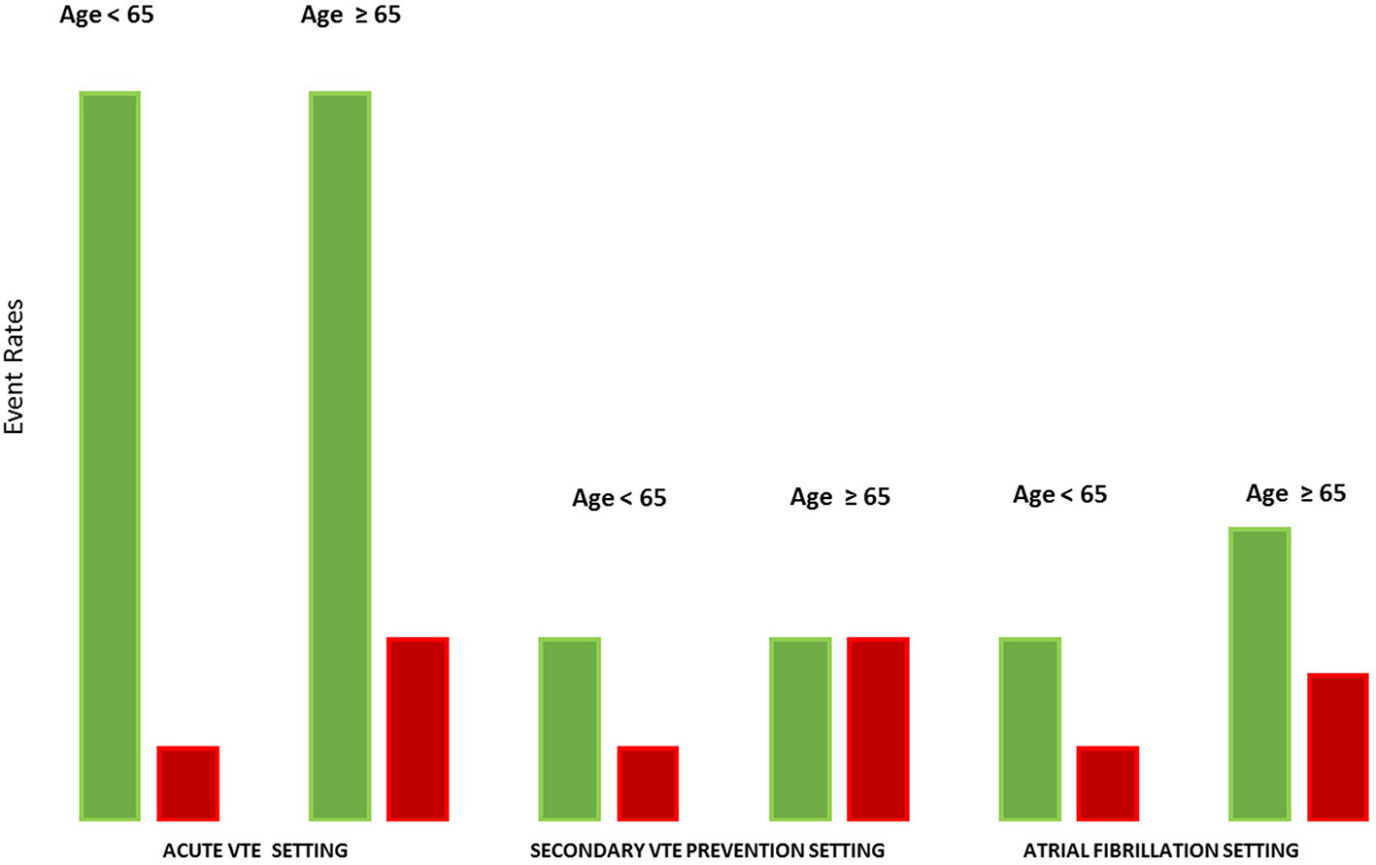
The dynamic between thromboembolic and bleeding risks according to age in various settings. The figure shows the dynamic between thromboembolic and bleeding risks according to age and to clinical indications. In the acute VTE setting, without anticoagulant therapy, the risk of recurrent VTE is very high irrespective of age. Although bleeding risk on anticoagulation increases with age, anticoagulant therapy is associated with a net clinical benefit in acute VTE treatment in younger and older patients. In the secondary VTE prevention setting, the risk of VTE recurrence after a treated index event is lower compared to the acute VTE setting and similar in both younger and older patients. Because of higher bleeding risk, the benefit of anticoagulation for secondary VTE prevention is likely reduced in older patients compared with younger patients. Consequently, VTE guidelines are less strong in recommending extended anticoagulation in older patients. By contrast, the risk of cardioembolic stroke in AF rises with age and thus most older patients continue to benefit from anticoagulant therapy despite a higher bleeding risk. Despite the similar definition of major bleeding, the consequence of a venous thromboembolic event and an arterial thromboembolic event are not equal. **Green**, thromboembolic risk in absence of anticoagulant therapy; **Red**, major bleeding risk with anticoagulation.

Up to 50% of patients with VTE have no identifiable risk factors and are classified as having unprovoked VTE. Such distinction is important because in general, patients with unprovoked VTE have higher lifetime risk of VTE recurrence after discontinuing anticoagulant treatment, with the risk of recurrence being at least 10% at 1 year and 30% at 5 years (75). The case fatality rate of a recurrence is 11% (76). The rate of recurrent VTE declines over time after the index event (77). This is a key distinguishing feature between VTE and SPAF in the elderly (Figure 2). Age remains a risk factor for anticoagulation-related bleeding in VTE patients. Thus, although the risk of VTE increases with age, guidelines have less strongly recommended extended anticoagulation in the elderly than in younger patients.

### VTE Treatment: The elderly in DOAC Trials of VTE

The four major DOAC VTE treatment trials (78–81) randomized patients to low molecular weight heparin (LMWH) bridging to VKA, or to DOAC with or without initial treatment with LMWH. The median age of subjects in these studies was between 55 and 60 years of age; the edoxaban study (82) reported that about 15% of patients were aged ≥75. Thus, the number of elderly patients represented in these randomized trials in acute VTE treatment was 3,294, which was less than in the SPAF trials. In those ≥75 years old, compared with VKAs, DOACs reduced recurrent VTE by 45% and major bleeding by 61% (83). A real-world study (84) reported outcomes of recurrent VTE and bleeding in over 12,000 patients on rivaroxaban and apixaban; 35% of the subjects were aged ≥65. Crude rate of major bleeding was about 2-fold higher in those ≥65 years old, but recurrent VTE was not more common in older patients. Although the results are reassuring, it is unclear how many very elderly patients were included.

### Practical Considerations in Dosing DOACs for VTE Treatment in the Elderly

VTE treatment is divided into initial (first week after the event), long-term (next 3 months after the event), and extended (3 months to indefinite) periods (77, 85). In the four major VTE treatment trials evaluating the DOACs for initial and long-term treatment, DOAC doses were not adjusted for age. The edoxaban study lowered the dose for subjects under 60 kg and with a CrCl between 30 and 50 mL/min (17% of the subjects), thus age was an indirect factor in dose reduction in this study, and subjects receiving low dose edoxaban had a similar benefit. Thus, in the absence of data, elderly patients with acute VTE treated with DOACs usually receive the standard doses initially. But, given that lower doses of apixaban and rivaroxaban have been validated as being effective and with a trend to less clinically relevant bleeding in extended treatment of unprovoked VTE and VTE provoked by minor risk factors (81, 86), it seems reasonable to consider these lower doses in elderly patients who need or prefer extended anticoagulation to prevent recurrent VTE.

## Conclusion

Both arterial and venous thromboembolism are more common in older adults, but so is the risk of anticoagulant-related bleeding. Because the risk of recurrent venous thrombosis decreases after the index event, unlike the persistent bleeding risk associated with extended anticoagulation, stopping anticoagulant therapy for secondary VTE prevention in some older adults can be considered. However, the risk of stroke in AF continues to increase with age and most older patients with AF benefit from continuing anticoagulant therapy. Although preventing stroke in AF has huge social and health economic benefits, older adults with AF remain undertreated despite the introduction of the DOACs. Bleeding remains an important complication of anticoagulation that contributes to under treatment in older patients at risk of thrombosis. Consequently, there is an unmet need for safer anticoagulation therapy. Trials are now underway to examine whether newer DOACs inhibiting FXI or FXII will be effective and safer.

## Author Contributions

PG and NC wrote and edited the manuscript. Both authors contributed to the article and approved the submitted version.

## Conflict of Interest

PG has received consulting fees from Bayer, Bristol-Myers-Squibb, Pfizer, Leo Pharma, Servier Canada and Valeo Pharma. NC reports a speaker fee from Bayer outside the submitted work.
